# Dietary methionine depletion and hydrogen sulfide‐producing genes in perivascular adipose tissue of male Wistar rats

**DOI:** 10.14814/phy2.70702

**Published:** 2025-12-19

**Authors:** Adam Corken, Davis Lee, Elizabeth C. Wahl, James D. Sikes, Keshari M. Thakali

**Affiliations:** ^1^ Department of Pediatrics University of Arkansas for Medical Sciences Little Rock Arkansas USA; ^2^ Arkansas Children's Research Institute Little Rock Arkansas USA

**Keywords:** arterial contractility, high‐fat diet, hydrogen sulfide, methionine restriction, perivascular adipose tissue

## Abstract

Perivascular adipose tissue (PVAT) regulates vascular tone, and high‐fat diets reportedly lead to the loss of its anti‐contractile properties. Methionine restriction recapitulates many caloric restriction metabolic effects and increases liver expression of activating transcription factor 4 (ATF4) and downstream targets with anti‐contractile properties such as cystathionine‐β‐synthase (CBS) and cystathionine‐γ‐lyase (CSE). We hypothesized that dietary methionine restriction would prevent high‐fat diet‐induced PVAT dysfunction by increasing ATF4 expression. Male Wistar rats were fed control (C, 10% calories from fat) or high‐fat diets (H, 60% calories from fat) (HFD) that were methionine replete (R, 0.86% methionine) or deplete (D, 0.12% methionine) for 12 weeks. Methionine restriction prevented body mass increases, independent of fat content, and had no effect on blood pressure or arterial contraction to norepinephrine (10 μM) or relaxation to acetylcholine (10 μM). In this feeding paradigm, HFD did not induce PVAT dysfunction. CR diet increased ATF4 transcript levels in multiple PVAT depots but did not consistently affect expression of downstream targets. Protein expression of ATF4 and its targets varied largely for all groups. In conclusion, 12 week methionine restriction profoundly affected body mass but not blood pressure or arterial reactivity in the presence or absence of PVAT.

## 1 INTRODUCTION

According to the World Health Organization and the Centers for Disease Control and Prevention (CDC), cardiovascular disease (CVD) is the leading cause of death worldwide and in the United States. A key contributor to the onset and progression of CVD is excessive weight gain causing an overweight or obese status (Koliaki et al., 2019; Powell‐Wiley et al., 2021). In 2022, the CDC reported that 73.6% of US adults were overweight (including those with obesity), thereby increasing the risk of CVD within the general populace to alarmingly high levels. Of note, it was also reported that 58% of those with obesity had high blood pressure or were taking medication to treat hypertension. Hypertension directly contributes to elevated risk for specific CVD maladies such as coronary artery disease, stroke, and heart failure (Díez & Butler, 2023; Howard et al., 2025; Nakanishi et al., 2017). While the CVD‐obesity‐hypertension axis has been extensively investigated to date, there are still many gaps in our knowledge regarding cellular crosstalk and molecular underpinnings of the CVD‐obesity‐hypertension relationship worth exploring to mitigate the deleterious impact these conditions have on human health and wellbeing.

It has long been understood that maintaining blood pressure homeostasis involves integration of endocrine signaling from neuronal, hepatic, and renal tissues (Shahoud et al., 2025). However, a more recent discovery has also demonstrated a specific adipose depot to be a key regulator of vascular tone. Until recently, PVAT, the adipose tissue surrounding blood vessels, was believed to be a structural component of the vascular stroma, but a series of investigations revealed it functions as a vital paracrine regulator of the endothelium and vascular smooth muscle (VSM) (Huang Cao et al., 2017; Soltis & Cassis, 1991). Under normal conditions, PVAT regulates vascular tone through the release of several anti‐contractile, soluble mediators that act upon the endothelium and VSM to facilitate vasorelaxation (Hillock‐Watling & Gotlieb, 2022). This relaxed vessel status is specifically orchestrated by PVAT‐derived hydrogen sulfide (H_2_S) production (Fang et al., 2009; Gheibi et al., 2018; Streeter et al., 2013). The anti‐contractile properties of H_2_S stem from its stimulation of ion channels present on endothelial and VSM cells, causing hyperpolarization and relaxation of blood vessels (Köhn et al., 2012; Mustafa et al., 2011; Wang et al., 2015). Similar to other adipose depots, PVAT can be modified by diet, with obesogenic diets significantly altering the phenotype and function of PVAT in animal models. Obesity confers an increase in oxidative stress within PVAT, causing secretory dysregulation and functional disruption resulting in vasocontraction, with reduced H_2_S production highlighted as an underlying cause (Bełtowski, 2013; Katsouda et al., 2018; Whiteman et al., 2010). This posits PVAT and H_2_S as an intriguing node within the CVD–obesity–hypertension axis as it provides a means by which diet/obesity directly influences blood pressure and enhances CVD risk. Despite this, there are significant knowledge gaps related to the mechanisms by which obesity and dietary factors affect vascular PVAT and H_2_S levels.

Interestingly, studies involving dietary reduction of the sulfur‐containing amino acid methionine yielded a number of metabolic benefits similar to caloric restriction (Ables et al., 2012), notably improved obesity burden by increasing fat oxidation (Plaisance et al., 2011), adipose tissue autophagy (Cooke et al., 2020), and increased overall resistance to high‐fat diet (HFD)‐induced onset of obesity (Ables et al., 2012; Wang et al., 2020). Moreover, further evidence indicates that restricting dietary methionine activates the integrated stress response (ISR) pathway, resulting in decreased general protein synthesis and preferential transcription of activating transcription factor ATF4 gene targets, such as CBS and CSE (Dickhout et al., 2012; Jonsson et al., 2019; Pettit et al., 2017; Stone et al., 2021). As CBS and CSE are capable of synthesizing H_2_S, methionine restriction provides a potential route by which diminished levels of H_2_S can be increased and, in turn, alleviate the vascular constriction/hypertension associated with obesity. Though a few studies assessed PVAT in the context of methionine restriction, the primary focus was to elucidate the ability to restore the “beige” phenotype (Hildebrand et al., 2018) of the tissue to improve either venous graft remodeling or age‐related effects of lipid profiles (Kip et al., 2024; McGilvrey et al., 2023). However, the functional aspects of PVAT as a chief regulator of vascular tone were unreported under these conditions of methionine restriction. As such, we hypothesized that methionine restriction to counteract the adverse vascular conditions of a HFD (a known driver of obesity) through ISR activation and subsequent increase of H_2_S producing enzymes. In the following study, we administered a methionine‐depleted diet on either control diet or HFD to male Wistar rats for 12 weeks. Wistar rats were specifically chosen due to their susceptibility to hypertension (Tukhovskaya et al., 2024). Blood pressure was monitored weekly, and at the study's conclusion, PVAT was isolated for functional assessment and protein expression. Additionally, the assessment of methionine‐replete control diet and HFD groups occurred in tandem. The results of the study are as follows.

## 2 MATERIALS AND METHODS

### 2.1 Rat model and diet administration

Male 5‐week‐old Wistar rats were purchased from Charles River and acclimatized for 1 week before being randomized to their specified diet. The rats were randomized to one of four diets: methionine replete control diet (CR, A11051302B Research Diets Inc., 0.86% methionine, 10% calories from fat, *N* = 10); methionine depleted control diet (CD, A11051301B Research Diets Inc., 0.12% methionine, 10% calories from fat, *N* = 10); methionine replete high‐fat diet (HR, A11051306 Research Diets Inc., 0.86% methionine, 60% calories from fat, *N* = 10); or methionine depleted high‐fat diet (HD, A11053105 Research Diets Inc., 0.12% methionine, 60% calories from fat, *N* = 10) for 12 weeks and were allowed ad libitum access to food and water. This resulted in four groups (CR, CD, HR, and HD). Rats were singly housed in opaque (polypropylene) cages stored on stainless steel racks in a ventilated, climate‐controlled room within the Arkansas Children's Research Institute's animal housing facility. Weight and food intake were assessed once a week. Twelve weeks after respective diets, all rats were euthanized via inhalation of CO_2_ followed by exsanguination. Serum, liver, adipose tissues (white and brown), thoracic and abdominal aorta, and the mesenteric bed were collected for further analyses. Serum was collected for leptin, insulin, glucose, triglycerides, total cholesterol, and nonesterified fatty acids quantification further described in the following section. PVAT was resected from the isolated thoracic and abdominal aortas shortly after sacrifice. The thoracic (tPVAT) and abdominal PVAT (aPVAT) were snap frozen in liquid nitrogen along with the liver, brown and white adipose tissues and then stored at −80°C. The Institutional Animal Care and Use Committee at the University of Arkansas for Medical Sciences approved all experimental procedures.

### 2.2 Body composition, fasting glucose tolerance testing, and serum analysis

Body composition was measured by EchoMRI (ECHO, Houston, TX, Model # EMR‐035) before randomizing to respective diets, and at 6 and 12 weeks after their respective diets. Glucose tolerance tests were performed before randomizing to respective diets, and at 5 and 11 weeks after being on their respective diets. Rats were fasted for 15 h prior to an intraperitoneal injection of 2.0 g/kg of glucose (PHR1000‐1G, Sigma Aldrich) dissolved in sterile saline. Two hours lapsed between the baseline (0 min) sample collection and the intraperitoneal injection of 2.0 g/kg of glucose (Sigma Aldrich, PHR1000‐1G). Samples were collected at 0, 15, 30, 60, and 120 min intervals following glucose injection. Glucose levels were determined using a blood glucose monitor (Bayer Contour Next Meter).

Serum samples were utilized to detect various analytes to discern the metabolic status of each cohort. Serum lipid profiles for triacylglyceride (TAG), nonesterified fatty acid (NEFA), and cholesterol were derived from Fujifilm biochemical colorimetric kits (LabAssay Triglycerides 291‐94501; LabAssay NEFA 299‐94301; LabAssay Cholesterol 293‐93601). Insulin and leptin were determined using MSD multi‐array kits (Insulin U‐PLEX K1536HK; leptin U‐PLEX K1535ZK). Glucose was calculated using an Infrared Laboratory Systems biochemical colorimetric assay (IR070).

### 2.3 Arterial blood pressure measurements

Arterial blood pressure was measured using tail cuff plethysmography using the CODA™ Monitor (Kent Scientific) noninvasive blood pressure monitoring system once every week until the end of the study. Prior to the initial assessment following the first week of diet administration, rats were acclimated to the tail cuff plethysmography procedure on three different days during the previously described acclimatization week following vendor delivery. Briefly, rats were secured in a nose cone holder and placed on a warming pad (maximum of 45°C) to cause blood vessel dilation. The cuff was placed on the tail and inflated automatically via the CODA instrument to occlude blood flow. The placement of an additional sensor recorded systolic and diastolic blood pressure upon deflation of the occluding cuff.

### 2.4 Mesenteric artery contractility

Following isolation of the mestenteric bed, 2nd order MAs were further isolated from the tissue. 2 mm sections from the one MAs were dissected with the surrounding PVAT removed from one section and maintained on the other before resection and mounting between two 45 μm wires in a myograph chamber (Danish Myo Technology, 620 M). Optimal passive tension was determined using the DMT normalization module that determines the internal circumference at which the vessel would be stretched to the equivalent of a transmural pressure of 100 mmHg. The myograph chamber was filled with warmed (37°C), oxygenated (95% O_2_, 5% CO_2_) physiological salt solution (in mM: NaCl,130; KCl, 4.7; KH_2_PO_4_, 1.18; MgSO_4_ × 7H_2_O, 1.17; CaCl_2_, 1.6; NaHCO_3_, 14.9; dextrose, 5.5; CaNa_2_EDTA, 0.03). After pulling passive tension and a 30‐min equilibration period, a tissue “wake up” was elicited by administering KCl of a final concentration of 60 mM to recapitulate active vessel tension akin to physiological conditions. After washing out the KCl solution, tissue viability (i.e., constriction/relaxation) was then assessed with a bolus challenge of 10 μM norepinephrine (NE) (DL‐Norepinephrine Hydrochloride, Sigma Aldrich A7256‐1G) followed by 10 μM acetylcholine (ACh) (Acetylcholine Chloride, Sigma Aldrich A9101‐10VL). The maximum contractile forces for NE and ACh were normalized to the maximum contractile force elicited by the KCl “wake up”. The normalized NE values were representative of contractile force following agonist stimulation while ACh were further normalized relative to NE values to determine the reduction in contraction (relaxation) resulting from antagonist treatment. Contractility data are represented as mean ± SEM.

### 2.5 Indirect calorimetry

Energy expenditure (EE) was measured at 1, 5, and 11 weeks on diets using the Promethion Indirect Calorimetry System (Sable Systems International, North Las Vegas, NV). The rats were singly housed in their home cages for 72 h within an environmentally climate‐controlled chamber (temperature, light, humidity, and other variables can be controlled). Values were recorded every minute, and these intervals were averaged into hourly data points. Circadian patterns were divided into two 12‐h periods with the dark cycle proceeding from 19:00 to 7:00 and the light cycle subsequently occurring from 7:00 to 19:00. All energy expenditure values were normalized to paired body mass measurements for each period of observation.

### 2.6 Real‐time quantitative PCR

Five hundred ng of total RNA was reverse‐transcribed using the iScript cDNA synthesis kit (Biorad, Hercules, CA 1708891). PCR amplification of 5 ng of cDNA was performed using 2X SYBR green master mix (FAST SYBR Green Master Mix, Applied Biosystems, Foster City, CA 4385612). PCR reactions were monitored using an ABI Prism 7500 Fast sequence detection system (Applied Biosystems). Gene‐specific primers were designed using Primer Express™ Software (Applied Biosystems). Relative amounts of mRNA were quantitated using a standard curve and normalized to the expression of β‐actin mRNA. Primer sequences can be found in Table S1.

### 2.7 Western blot analysis

100 mg of liver, tPVAT and aPVAT samples from each dietary group were thawed on ice for 30 min. RIPA buffer containing a protease and phosphatase cocktail (Halt Protease & Phosphatase Inhibitor cocktail 100×, Thermo Fisher 78,440) was added to each sample tube followed by mechanical disruption with a Precellys Homogenizer (Bertin Technologies) for 1 min. Samples were incubated on ice for 30 min and then centrifuged at 15,000 rcf for 15 min at 4°C. Protein lysate supernatant was collected from each sample tube and the concentration was determined with a BCA assay kit (Pierce BCA Protein Assay, Thermo Fisher 23225).

30 μg of protein was loaded into a precast 4%–12% gradient Bis‐Tris gel (Thermo Fisher). Following electrophoresis resolution, the protein content of the gel was transferred to a 0.45 μM PVDF membrane (Bio‐Rad). After transfer, the membrane was incubated with blocking buffer (Tris Buffered Saline with 0.1% Tween 20 [TBST] and 5% BSA) for 1 h at room temperature with gentle agitation. The membrane was then washed three times with TBST for 5 min and then incubated overnight at 4°C with primary antibody diluted in blocking buffer. The membrane was again washed with TBST three times and treated with a secondary antibody diluted in blocking buffer for 30 min at room temperature. Following secondary antibody staining, the membrane was washed with TBST three times and treated with HRP substrate (Thermo Fisher, Thermo Scientific SuperSignal West Femto Substrate 34095) and imaged on iBright Imaging System (Thermo Fisher).

The following primary antibodies and dilutions were utilized in our analysis; GAPDH (Cell Signal Technologies 2118S) 1:1000, CBS (Cell Signal Technologies 14782) 1:1000, ATF4 (Cell Signal Technologies 11815) 1:1000, CSE (ProteinTech 12217‐1‐AP) 1:2500, UCP‐1 (Cell Signal Technologies 72298S) 1:1000, and leptin (Abcam ab16227) 1:1000. The following secondary antibody was used at a dilution of 1:1000, anti‐rabbit IgG, HRP‐linked (Cell Signal Technologies 7074S). Western blot quantitation was performed using iBright Analysis Software (Thermo Fisher) to derive relative quantities of protein by normalizing target pixel intensities to that of the GAPDH loading control.

### 2.8 Statistical analysis

A two‐way ANOVA was used to compare each measure variable between all groups, with a Tukey's post hoc comparison used to evaluate statistical differences between individual groups. In all cases, a *p* value less than or equal to 0.05 was considered to be statistically significant.

## 3 RESULTS

### 3.1 Dietary methionine depletion reduces body mass accumulation

**FIGURE 1:**
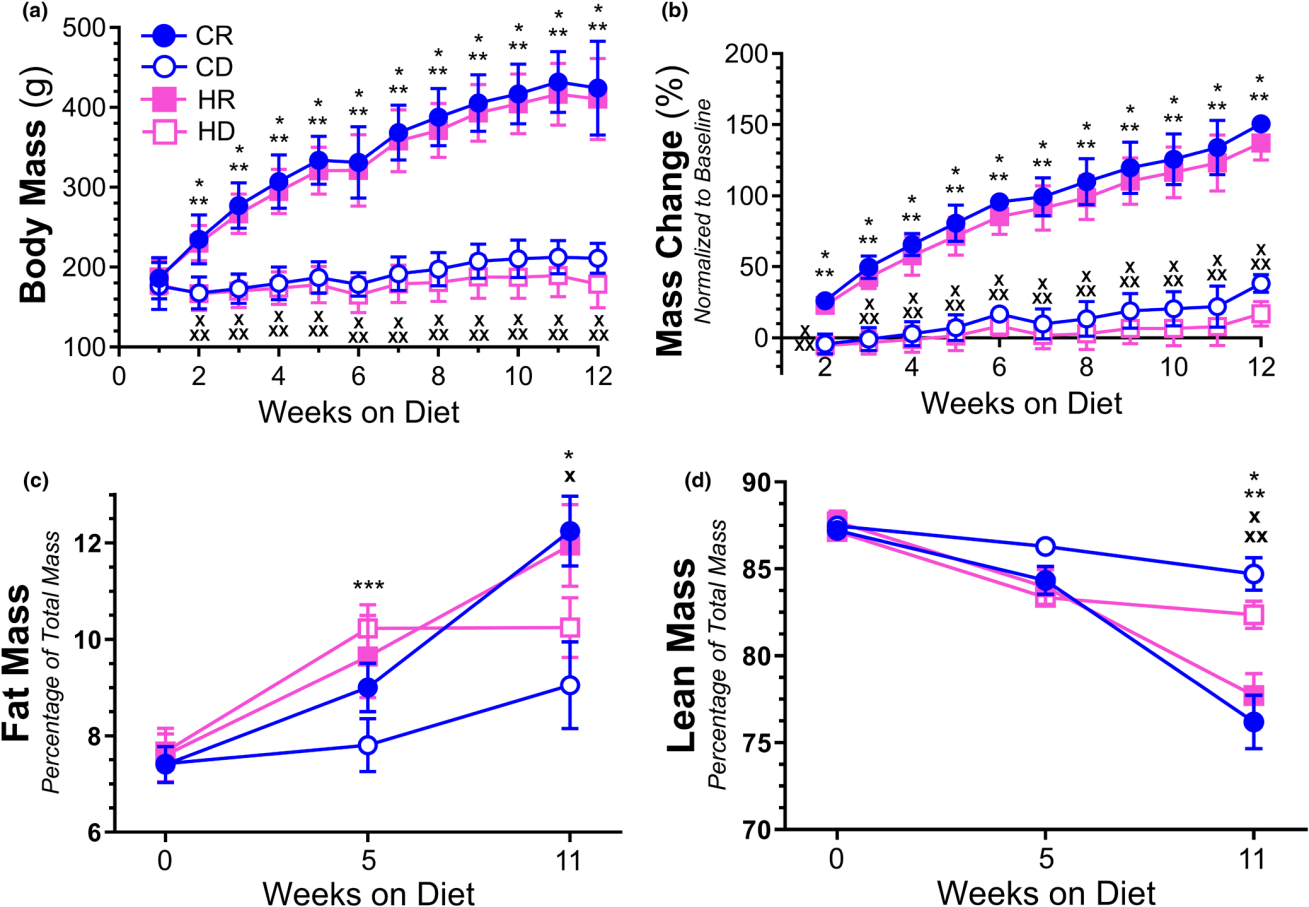
Body mass was measured once weekly from the onset of diet administration until the study's conclusion (a) *p* < 0.0001—*CR versus CD; **CR versus HD; ***CD versus HD; ^X^HR versus CD; ^XX^HR versus HD. The percent change in body mass was also normalized to initial body mass measurements (b) *p* < 0.0001—*CR versus CD; **CR versus HD; ***CD versus HD; ^X^HR versus CD; ^XX^HR versus HD. Fat mass (c) [*p* = 0.0022—*CR vs. CD; *p* = 0.0066—^X^CD vs. HR; *p* = 0.0320—***CD vs. HD] and lean mass (d) were assessed prior to and at 5 and 11 weeks of diet administration [*p* value < 0.0001—*CR vs. CD; **CR vs. HD; ^X^HR vs. CD; *p* value = 0.0007—^XX^HR vs. HD]. *N* = 10 per group.

The administration of both the methionine replete CR and HR diets resulted in substantial total body mass (g) accumulation throughout the duration of the 12‐week study (Figure 1a). Moreover, the CR and HR diet groups displayed similar body masses, on average, at all points of observation despite the diets differing in fat content (10% vs. 60% calories from fat). Interestingly, methionine depletion, regardless of diet fat content, elicited marginal body mass gains such that both CD and HD groups differed significantly from their methionine replete counterparts. As such, groups clustered based on methionine content and not dietary fat composition. When assessing the percentage of body mass change normalized to baseline values, again we saw clustering of groups based on dietary methionine content, with the methionine replete groups displaying significantly higher percent changes in body mass (Figure 1b). While the methionine replete groups demonstrated a ~150% change in body mass by the study conclusion, methionine deplete groups exhibited nominal (~10%–30%) changes. Assessment of fat mass composition revealed that fat mass as a percentage of total mass did not differ between all four groups at baseline (Figure 1c). After 5 weeks of diet administration, CD mice had significantly less fat mass relative to HD mice, but this difference was not observed after 11 weeks. Of note, the methionine replete groups, CR and HR, had elevated fat mass levels at 11 weeks when compared to their matched, methionine deplete counterparts. Measurements of lean mass composition showed no difference among all four groups at baseline and at 5 weeks of feeding (Figure 1d). However, by 11 weeks, both methionine deplete groups had substantially greater lean mass when compared to either methionine replete group. Total fat and lean mass measurements are available in Figure S1. Despite the observed delineations in total body mass and fat/lean mass resulting from dietary methionine content, glucose tolerance testing (GTT) did not yield significant differences in glucose processing at baseline, 5, or 11 weeks of dietary intervention (Figure S2). Additional analysis of metabolic analytes after 11 weeks of feeding indicated that methionine depletion significantly reduced nonfasting serum insulin (Figure S3a) and increased serum leptin levels (Figure S3b) when compared to methionine replete samples regardless of dietary fat content. Non‐fasting serum glucose was higher in HD samples relative to both control diet cohorts (Figure S3c), which differs from previous GTT assessments but could be the result of the serum collection during an unfasted state. Interestingly, methionine depleted groups displayed higher levels of non‐fasted triacylglycerol (TAG) relative to methionine replete samples (Figure S3d), though total cholesterol (Figure S3e) and nonesterified fatty acids (NEFAs) (Figure S3f) were comparable amongst all groups regardless of methionine or fat content.

### 3.2 Dietary methionine depletion increases energy expenditure

**FIGURE 2:**
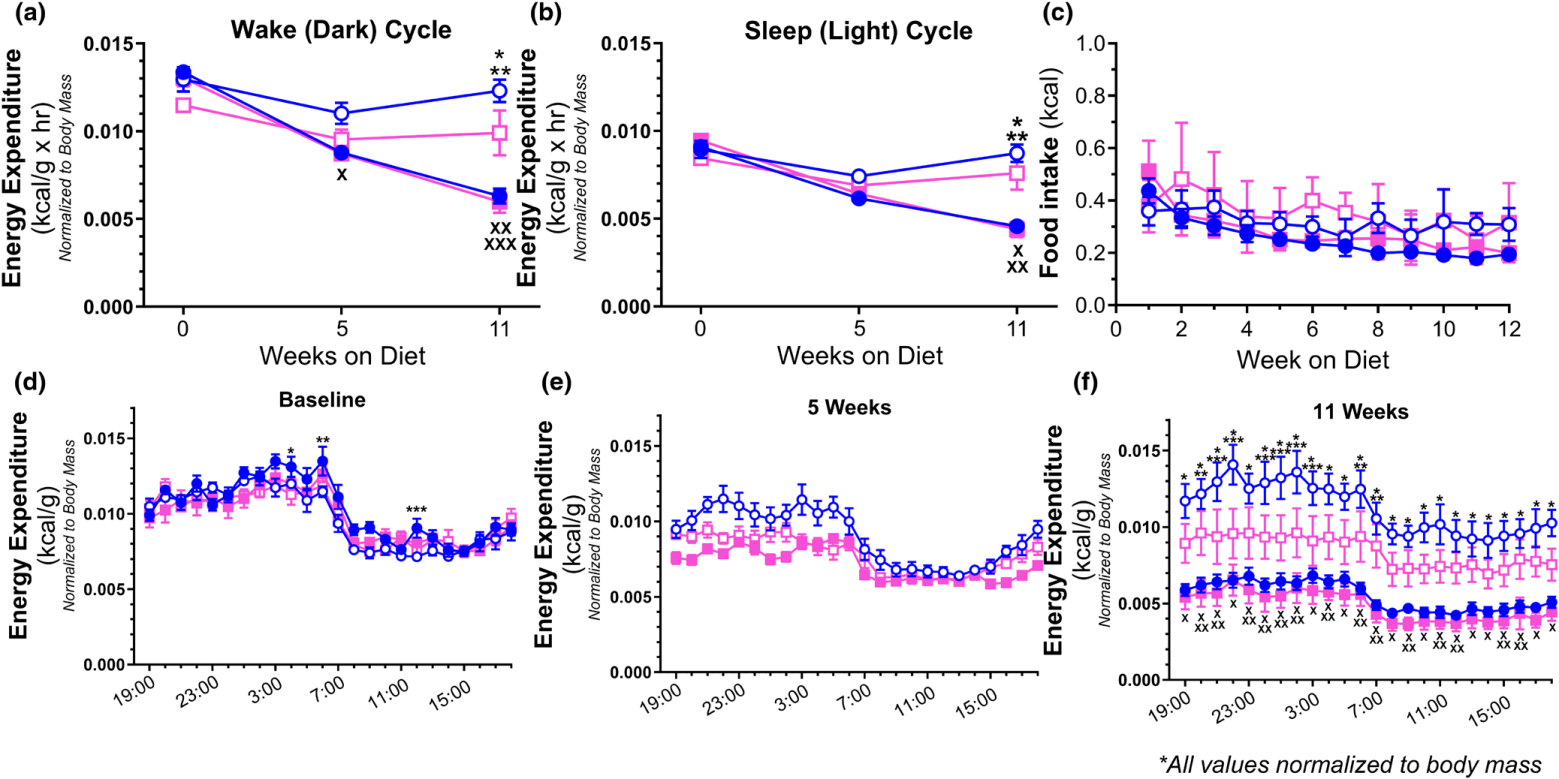
Average hourly energy expenditure normalized to body mass prior to and at 5 and 11 weeks following diet administration for 12 h periods during the dark cycle (19:00 to 7:00, (a) [*p* = 0.0456—^X^CD vs. HR; *p* = 0.0005—CR vs. HD; *p* < 0.0001—*CR vs. CD; **CD vs. HR; ^XX^HR vs. HD] and the light cycle (7:00 to 19:00) (b) [*p* < 0.0001—*CR vs. CD; **CR vs. HD; ^X^CD vs. HR; ^XX^HR vs. HD]. Average weekly caloric intake normalized to body mass (c). Average hourly energy expenditure normalized to body mass for a representative 24 h period prior to (d) [*p* = 0.0461—*CR vs. HD; *p* = 0.0218—**CR vs. CD; *p* = 0.0376 ****CR vs. CD] and following 5 weeks (e) and 11 weeks (f) [*p* < 0.05—*CR vs. CD; **CR vs. HD; ***CD vs. HD; ^X^HR vs. CD; ^XX^HR vs. HD] of diets. *N* = 10 per group.

Measuring EE through indirect calorimetry showed no difference in baseline EE values between groups when comparing average hourly kcal utilization normalized to body mass over a 12‐h period of activity/wakefulness (Figure 2a). After 5 weeks of diet consumption, the groups largely remained indiscernible, but at 11 weeks, both methionine‐depleted groups exhibited elevated dark cycle EE levels when compared to either methionine‐replete group. A similar pattern was observed when viewing EE during the 12‐h light cycle (Figure 2b). Overall, all groups displayed lower EE at all three time points during the light cycle relative to the dark cycle and were indistinguishable at baseline and after 5 weeks of feeding. However, at 11 weeks, both the CD and HD groups displayed markedly higher levels of EE when compared to either CR or HR, in a manner similar to the dark cycle observations. In consideration of food consumption variation possibly accounting for the differences observed in EE, we assessed weekly food intake relative to body mass (Figure 2c). We could not distinguish differences in food intake (normalized to body mass) between groups, thereby indicating the difference observed in EE was most likely due to dietary methionine content. The visualization of the individual hourly EE for each group over a complete 24‐h period at the baseline (Figure 2d), 5 weeks (Figure 2e), and 11 weeks (Figure 2f) time points demonstrated that typical circadian EE flux (higher dark cycle and lower light cycle EE) for each group was maintained regardless of diet at all recorded time points. Furthermore, the 24‐h EE profiles were similar among all groups at baseline and 5 weeks, with 11 weeks of feeding resulting in an elevation of EE of methionine‐deplete groups and depression of methione‐replete groups EE. Interestingly, the CD group demonstrated a trend of having higher average EE levels across the entire 24‐h period relative to the other methionine‐deplete (HD) group, indicating that dietary fat content in the context of methionine depletion may be a relevant influencer of EE with prolonged feeding.

### 3.3 Dietary methionine depletion had no effect on vascular reactivity

**FIGURE 3:**
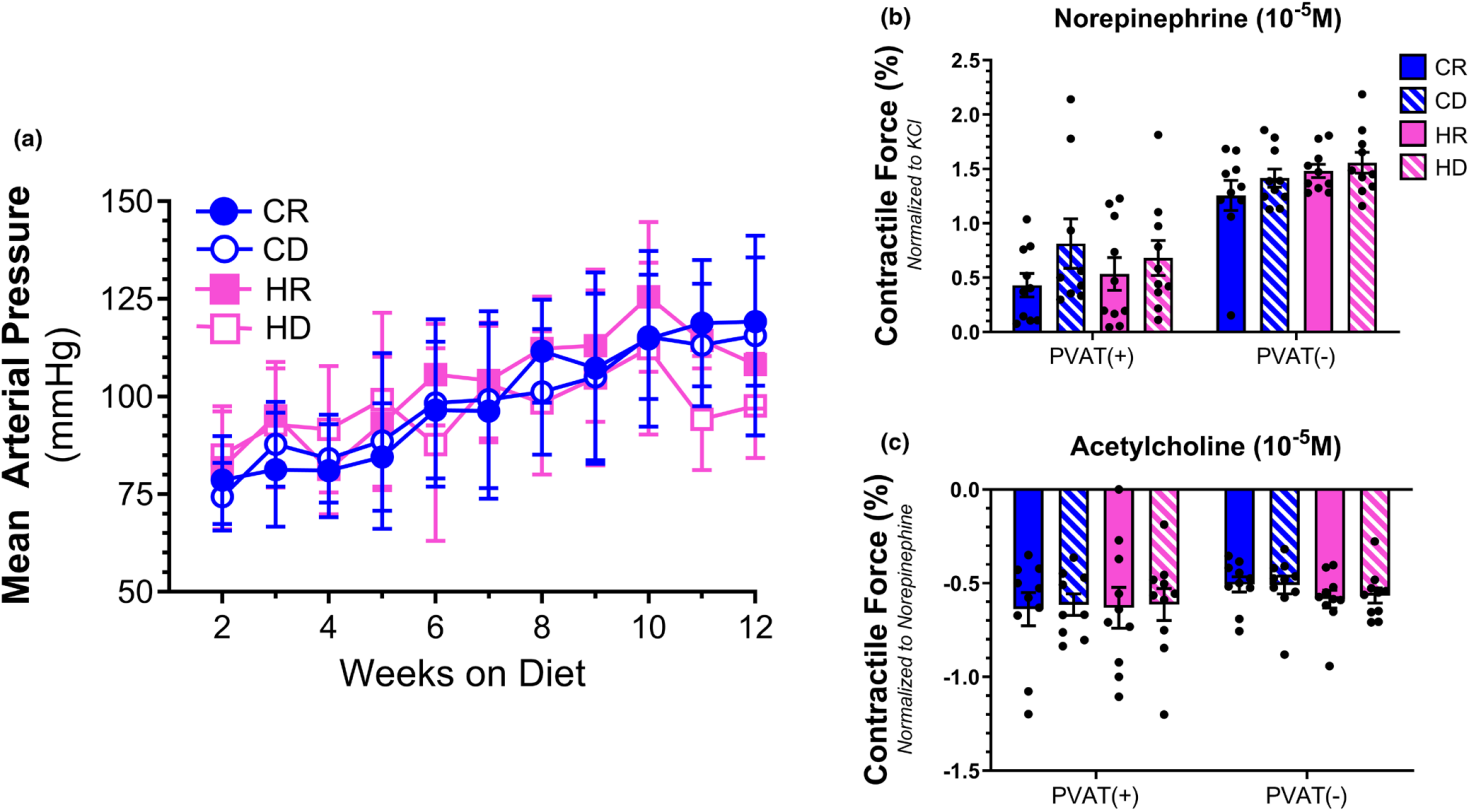
Mean arterial pressure was recorded weekly via the tail using a tail cuff monitoring system (a). Contraction to norepinephrine (b) and relaxation to acetylcholine (c) in NE‐precontracted mesenteric arteries with [PVAT (+)] or without PVAT attached [PVAT (−)] were normalized to baseline values generated during the initial tissue “wake up” induced by KCl administration. *N* = 10 per group.

Alterating dietary methionine content did not cause changes in mean arterial blood pressure when measured by tail cuff (Figure 3a). Similarly, dietary fat content also had no impact on arterial pressure. Of note, arterial blood pressure demonstrated a gradual increase over time for all groups regardless of diet. Systolic and diastolic pressures were likewise unaffected by dietary fat or methionine content (Figure S4). Investigation of the contractile properties of isolated mesenteric arteries revealed that neither dietary methionine nor fat content altered the contractile force output for the vessel with surrounding PVAT intact when challenged with the agonist NE (Figure 3b). The removal of surrounding PVAT resulted in an increase in the contractile force output for all groups when treated with NE. This heightened vessel contraction resulting from PVAT loss is a well‐documented phenomenon and did not lead to any stratification of groups based on dietary methionine or fat content. The administration of the vasodilator ACh led to a reduction in contraction following NE pre‐constriction, with all groups demonstrating similar levels of relaxation (Figure 3c). Likewise, arterial samples without PVAT exhibited relaxation to ACh and were indistinguishable from samples with intact PVAT. Furthermore, samples without PVAT were unaffected by dietary methionine or fat content, similar to those with maintained PVAT.

### 3.4 Methionine consumption does not elicit ISR alterations within aPVAT

**FIGURE 4:**
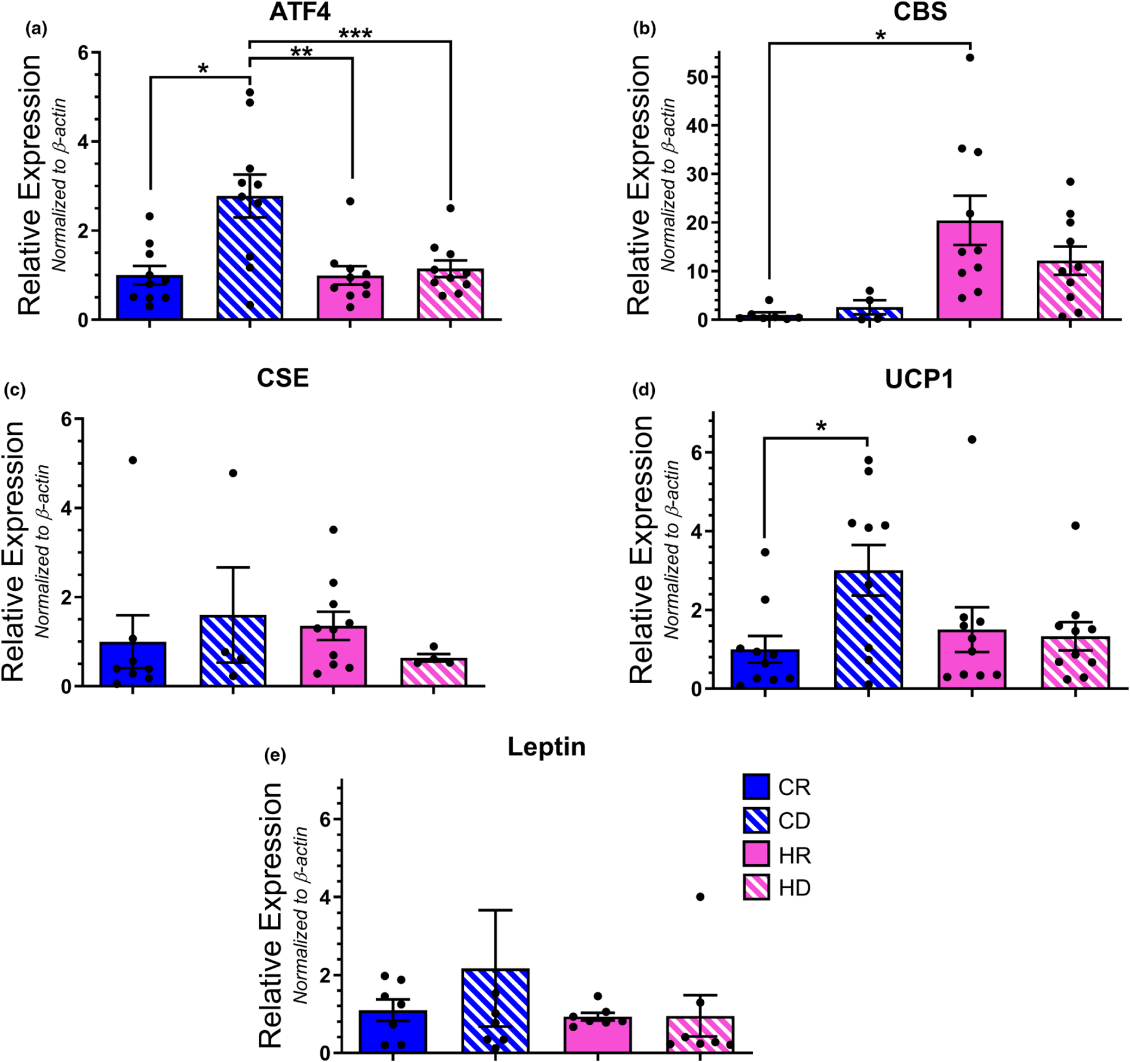
Transcript analysis of ISR member proteins in aPVAT samples. Relative expression levels for ATF4 (a) [*p = 0.0017; **p = 0.0016; ***p = 0.004], CBS (b) [*p = 0.0133], CSE (c), UCP1 (d) [*p = 0.0229], and leptin (e) mRNA were determined via qPCR analysis and normalized to β‐actin. *N* = 10 per group.

**FIGURE 5:**
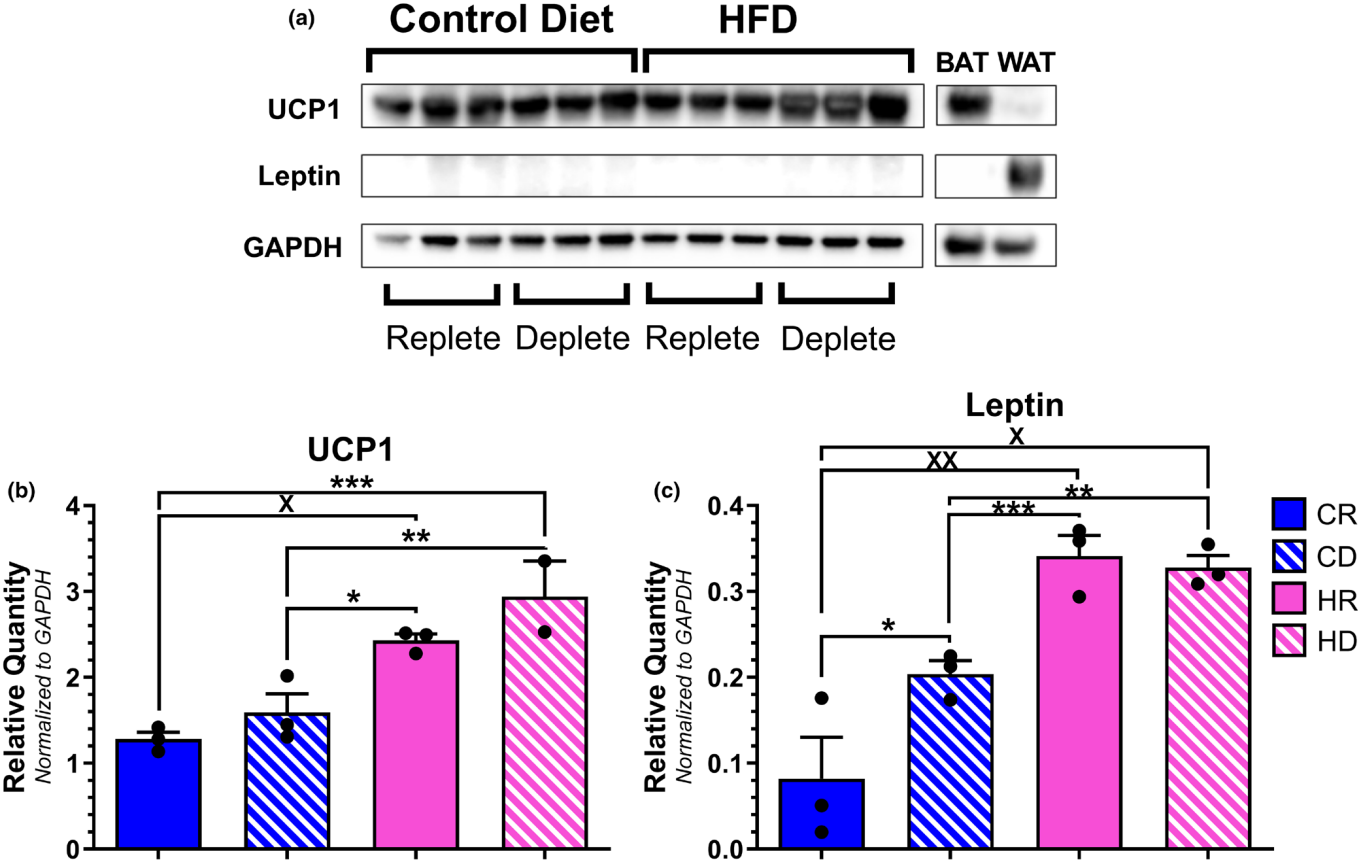
Western blot analysis of ISR and adipose tissue‐specific proteins in aPVAT, brown adipose tissue (BAT), and white adipose tissue (WAT) lysates (a). UPC1 (b) [*p = 0.0115; **p = 0.0032; ***p = 0.0410; ^X^ *p* = 0.0077] and leptin (c) [*p = 0.0411; **p = 0.0010; ***p = 0.0013; ^X^ *p* = 0.0244; ^XX^ *p* = 0.0379] densitometry values from each target were normalized to GAPDH. *N* = 3 per group. Complete images of aPVAT Western blot analysis is found in Figure S5.

We investigated the impact of dietary methionine restriction on downstream ISR targets within the aPVAT depot. Dietary methionine depletion significantly increased the transcription of ATF4 when coupled with a control diet alone (Figure 4a). When observing the downstream targets of ATF4, we observed a trend toward elevated CBS transcription in high‐fat diet cohorts independent of methionine content, though this failed to reach the level of significance (Figure 4b). Furthermore, neither fat nor methionine content elicited a change in CSE transcription (Figure 4c). Additionally, obesogenic conditions are known to facilitate the transition of PVAT from its default, anti‐contractile, beige phenotype to a dysfunctional, contractile, white phenotype. Caloric restriction has been shown to mitigate this transition and previous studies investigating methionine restriction posited that the effects of a low‐methionine diet mimicked those of caloric restriction (Lee et al., 2016; McCarty et al., 2009; Yu et al., 2018). As such, we also investigated whether methionine restriction affected aPVAT phenotype by observing brown/beige (Uncoupling Protein 1;UCP‐1) and white (leptin) adipose markers. An elevated mean expression of UCP1 transcript in CD samples indicates that methionine depletion paired with a control diet facilitated a brown/beige PVAT phenotype (Figure 4d). However, this observation did not reach statistical significance when referenced to most of the other cohorts. Moreover, leptin transcription was not impacted by either dietary fat or methionine content (Figure 4e). We then proceeded to match protein levels to our transcript observations (Figure 5). ATF4, CBS, CSE, and leptin were not readily detectable with visual analysis. Normalization of pixel density revealed UCP1 (Figure 5b) and leptin (Figure 5c) levels were significantly increased in both HFD groups when compared to the control diet cohorts. Thus, there was not a clear correlation between ISR member transcript and protein levels resulting from dietary methionine variation in aPVAT.

### 3.5 Dietary methionine restriction did not alter tPVAT downstream ISR gene expression

**FIGURE 6:**
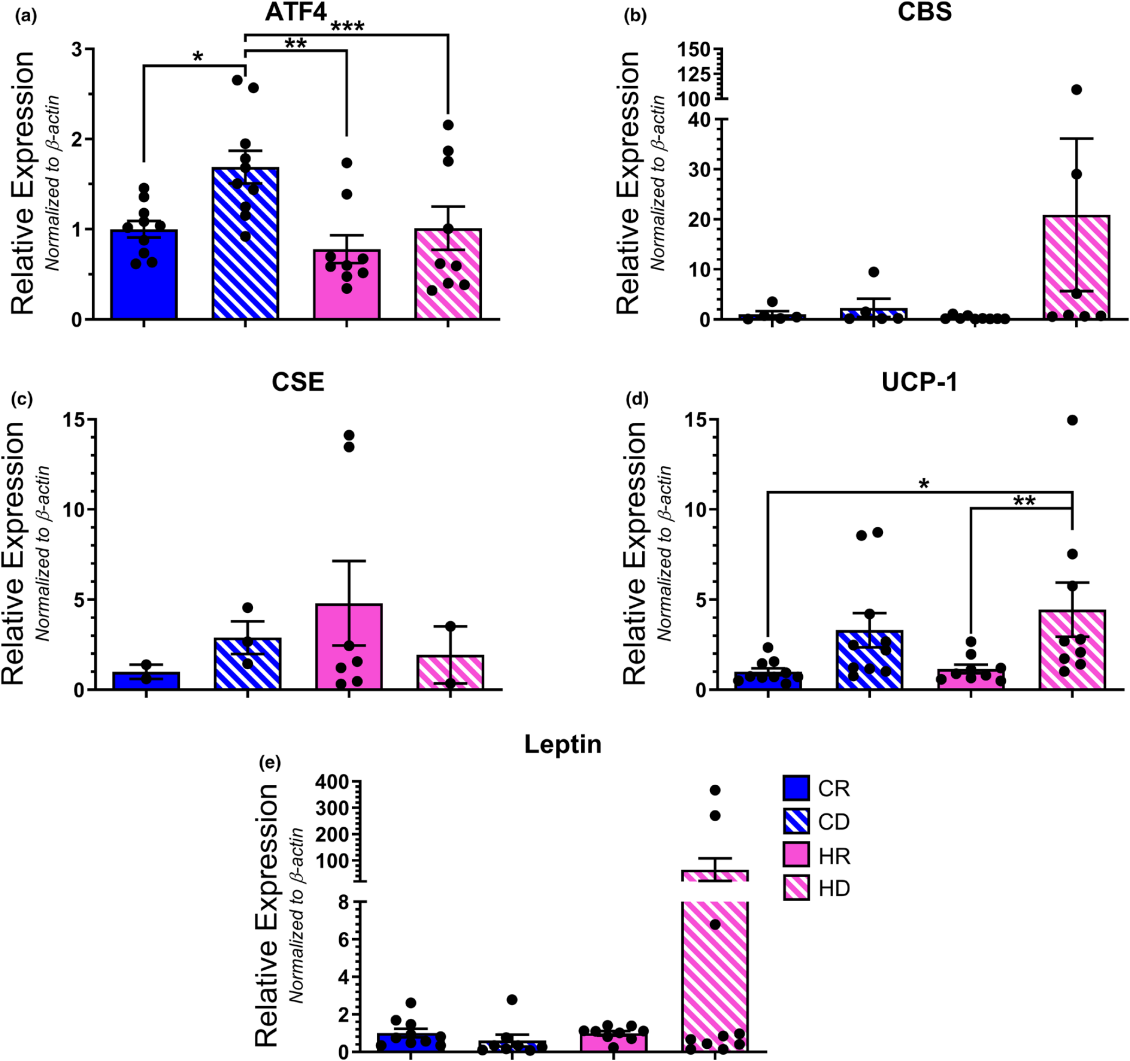
Transcript quantification of ISR and adipocyte‐specific protein within tPVAT samples. Relative expression levels for ATF4 (a) [*p = 0.0197; **p = 0.0023; ***p = 0.0179], CBS (b), CSE (c), UCP1 (d) [*p = 0.0139; **p = 0.0221], and leptin (e) mRNA were determined via qPCR analysis and normalized to *β*−actin. *N* = 10 per group.

**FIGURE 7:**
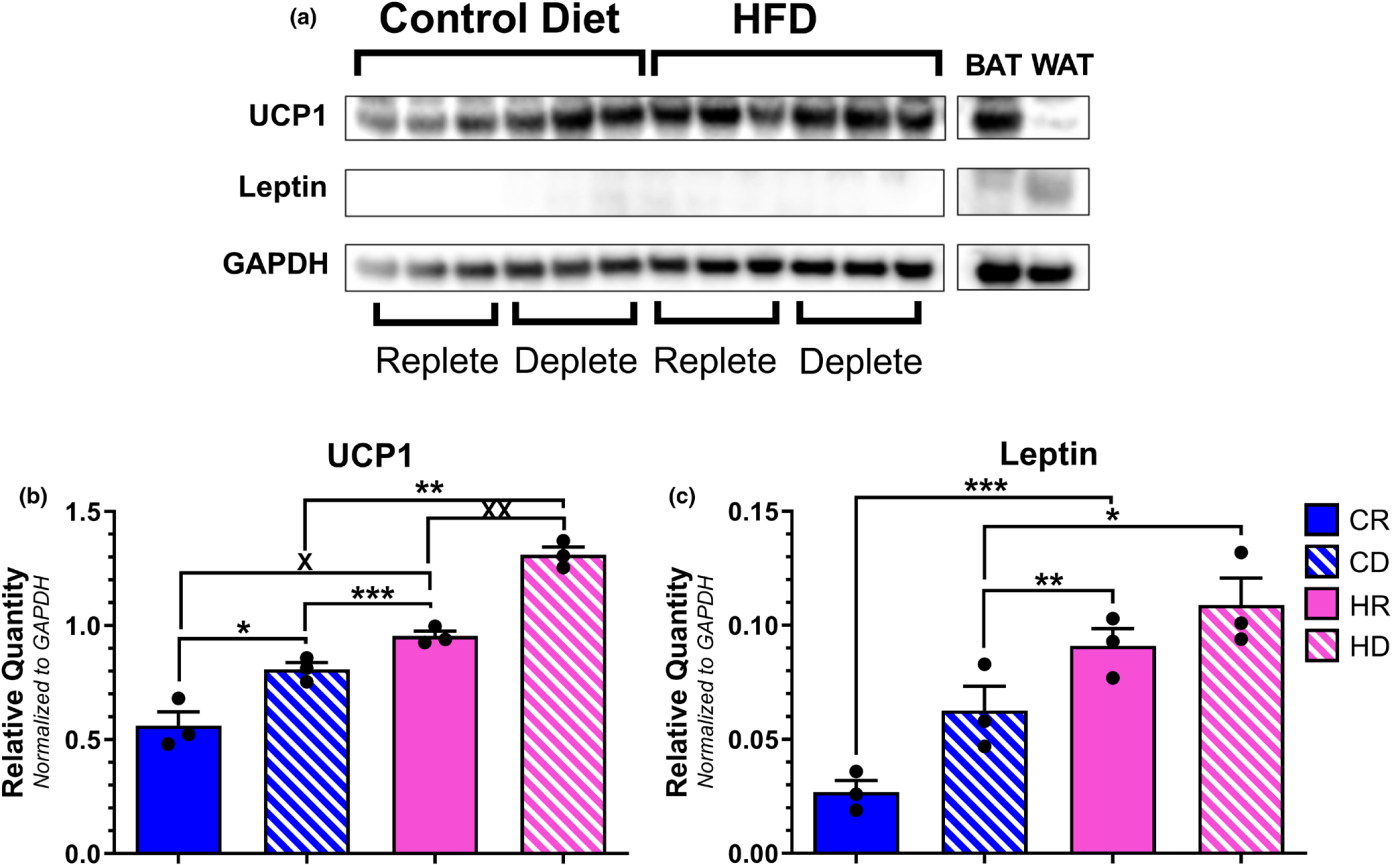
Immunoblot of tPVAT, BAT, and WAT lysates for ISR and adipocyte‐specific target proteins (a). UCP1 (b) [*p = 0.0230; **p = 0.0023; ***p = 0.0001; ^X^ *p* = 0.0006; ^XX^ *p* = 0.0039] and Leptin (c) [*p = 0.0090; **p = 0.0025; ***p = 0.0388] densitometry values for each target were normalized to GAPDH. *N* = 3 per group. Complete images of tPVAT Western blot analysis are found in Figure S6.

Similar to aPVAT, the CD group demonstrated markedly elevated ATF4 transcription within tPVAT relative to all others (Figure 6a). Despite this, CBS (Figure 6b) and CSE (Figure 6c) transcript levels were comparable between all groups. Of note, the HD group trended toward an increased mean expression of CBS, but this result was skewed by two data points within the sample population. Interestingly, methionine depletion appeared to upregulate UCP1 transcription as both the CD and HD groups on average displayed higher UCP1 expression (Figure 6d). It is worth noting that only the HD group was statistically different from both control cohorts. Lastly, leptin transcription did not differ when comparing groups, though the HD group trended toward a higher overall average due to higher variability in expression levels (Figure 6e). When assessing the protein expression of each target, ATF4, CBS, and CSE were undetectable in tPVAT upon initial visual inspection, similar to aPVAT. HD samples demonstrated the highest levels of UCP1 protein content within tPVAT when compared to all other groups, similar to the trend observed for transcript levels wherein HD UCP1 was higher than both methionine replete cohorts (Figure 7a,c). Leptin protein levels were decreased in CD tPVAT samples relative to both HFD groups, while CR leptin levels were only significantly reduced relative to HR samples (Figure 7b,c). And so, similar to the aPVAT analysis, when referencing qPCR to Western blot data, the observed transcript levels were largely incongruent with the perceived protein content.

### 3.6 Dietary methionine alters transcript and protein levels of ISR members in the liver

**FIGURE 8:**
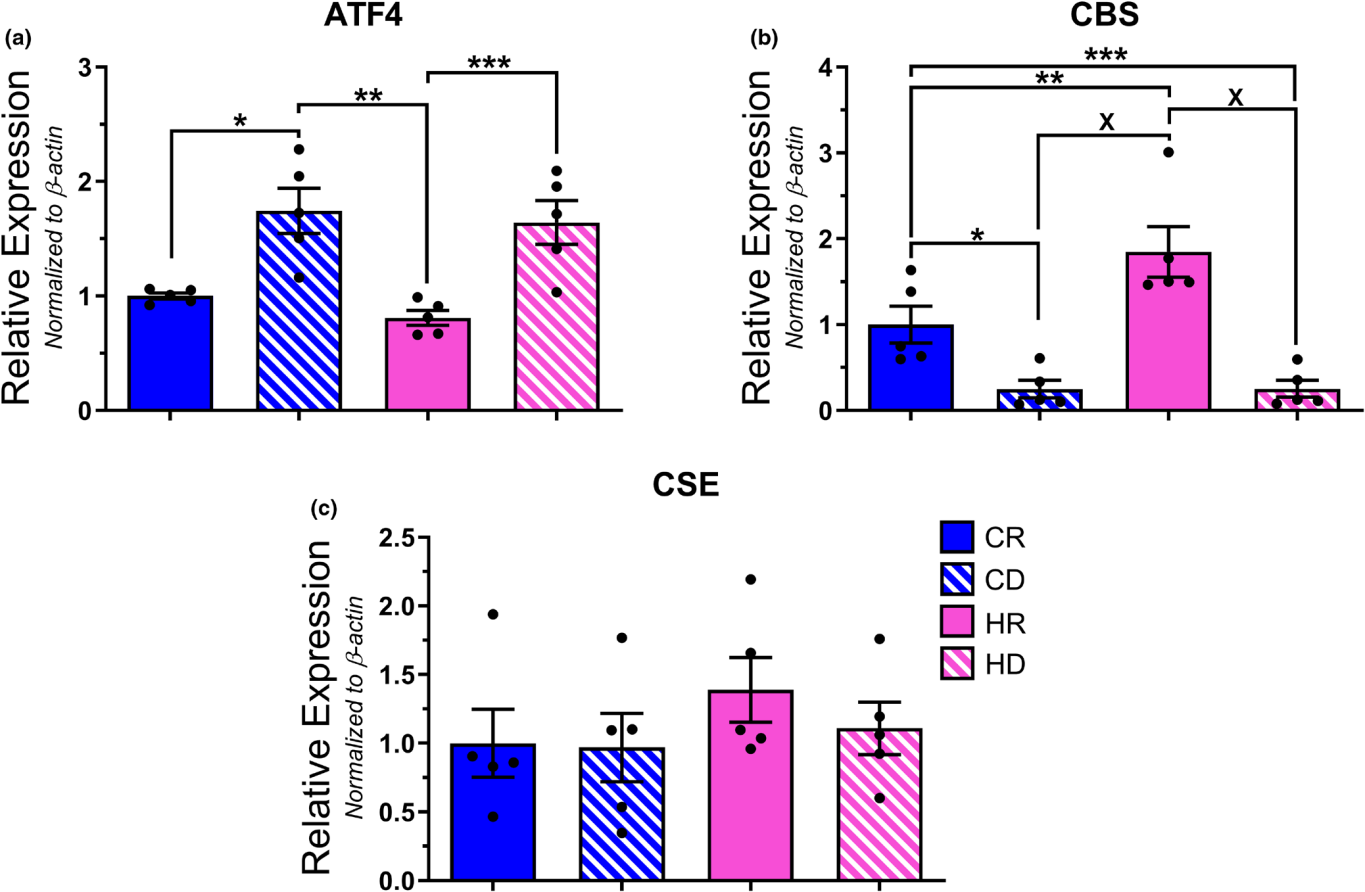
Determination of ISR family member transcript expression within liver samples. Relative expression levels for ATF4 (a) [*p = 0.0260; **p = 0.0058; ***p = 0.0129], CBS (b) [*p = 0.0447; **p = 0.0226; ***p = 0.0459; ^X^ *p* = 0.0002], and CSE (c) mRNA were determined via qPCR analysis and normalized to β‐actin. *N* = 10 per group.

**FIGURE 9:**
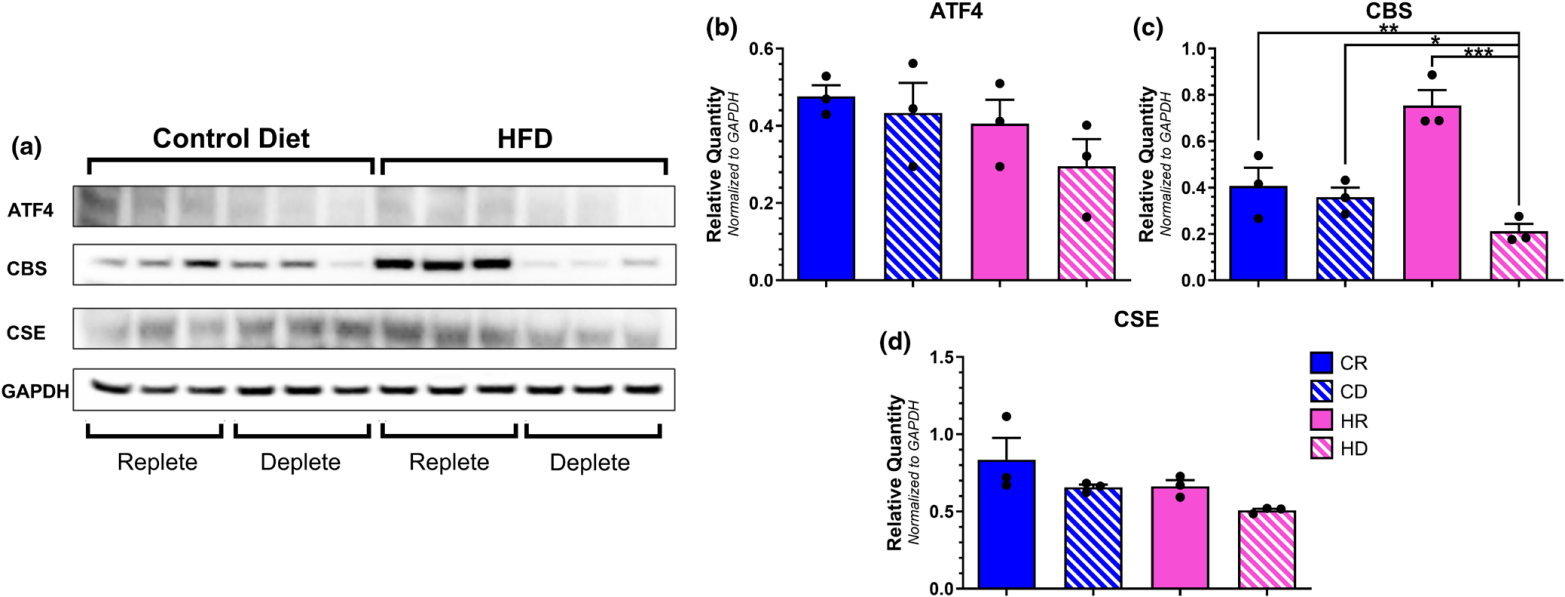
Western blot visualization of ISR members from liver lysates (a). ATF4 (b), CBS (c) [*p = 0.0358; **p = 0.0201; ***p = 0.0044] and CSE (d) densitometry values were normalized to GAPDH for each sample. *N* = 3 per group. Complete images of liver Western blot analysis are found in Figures S7 and S8.

As the liver is capable of producing mediators of vascular tone and likewise is influenced by the ISR, we investigated the transcript and protein levels of ATF4 and the downstream targets CBS and CSE (Liu et al., 2022; Ohkubo et al., 2022). When analyzing mRNA expression as determined by qPCR, we observed that methionine depletion, and not dietary fat content, increased ATF4 transcript levels (Figure 8a). Surprisingly, CBS mRNA was inversely correlated with that of ATF4 as the methionine depleted groups exhibited significantly reduced levels relative to those with replete dietary methionine content (Figure 8b). Additionally, within the methionine replete groups, higher dietary fat content resulted in markedly elevated CBS expression relative to the matched control. Despite the observed changes in ATF4 and CBS, CSE mRNA expression within the liver was unaffected by dietary methionine or fat content (Figure 8c). We next corroborated qPCR data with protein expression levels using Western Blots (Figure 9a). Unlike PVAT samples, ATF4, CBS, and CSE were detected upon initial visual inspection within liver samples. ATF4 protein levels did not exhibit a similar pattern to our transcriptional analysis as there was no difference observed between dietary groups (Figure 9b). On the other hand, CBS expression within HR samples was significantly elevated relative to all other groups which was comparable to the qPCR results (Figure 9c). CSE protein also followed a similar trend as seen in the transcriptional data as CSE expression was indiscernible amongst the groups regardless of diet administration (Figure 9d).

## 4 DISCUSSION

In this study, we investigated the effects of dietary methionine depletion or restriction on metabolic function, vascular physiology, and perivascular adipose tissue (PVAT) contractility in male Wistar rats with or without HFD. Our major findings demonstrate that methionine restriction, irrespective of dietary fat content, significantly reduced total and fat mass accumulation, increased energy expenditure (EE), and enhanced lean mass composition. However, methionine restriction did not influence arterial blood pressure or PVAT‐mediated vascular responses in mesenteric arteries. These findings expand on the growing body of literature suggesting that sulfur amino acid restriction induces favorable metabolic changes, while also raising important questions about its role in modulating vascular function.

Methionine depletion led to profound differences in body weight and adiposity, consistent with prior studies in both rodents and humans showing that methionine restriction mimics many of the metabolic benefits of caloric restriction (Elshorbagy et al., 2011; Fang et al., 2021; Latimer et al., 2018; Orentreich et al., 1993; Perrone et al., 2012; Swaminathan et al., 2021). Dietary methionine was a significant factor in determining the total body mass changes over time as the groups consuming a diet with preserved methionine content, regardless of dietary fat levels, significantly increased their weight throughout the duration of the study while the methionine‐depleted groups displayed marginal weight changes. Specifically, the maintenance of dietary methionine resulted in an increase in fat mass and a loss of lean mass while methionine depletion attenuated the severity of these trends. It was notable that both methionine‐replete groups displayed virtually identical changes in total mass and fat/lean mass even though the administered diets differed significantly in fat content. Additionally, observing bioenergetic parameters also illustrated the impact dietary methionine content exerts on whole body metabolism.

A methionine‐restricted diet has repeatedly been shown to increase energy expenditure through several mechanisms, mostly involving fat oxidation in the liver (Ables et al., 2012; Miller et al., 2005; Nagarajan et al., 2024; Orgeron et al., 2014; Wanders et al., 2017). While energy intake did not differ amongst all groups regardless of dietary fat or methionine content, energy expenditure was markedly shifted due to dietary fat content. The onset of energetic perturbations was not rapid, as energy expenditure remained comparable amongst all groups after 5 weeks of diet administration. Despite the overall similarities, there are signs of methionine depletion heightening energy expenditure during the dark cycle. These 5‐week trends further deviated to become significantly pronounced with continual diet consumption, as methionine depletion elevated energy expenditure during both the dark and light cycles to encompass the entire 24‐h period. Increased EE observed at 11 weeks, particularly during both dark and light cycles, supports earlier reports that methionine restriction promotes fat oxidation and elevates basal metabolic rate (Nagarajan et al., 2024). Notably, these metabolic effects occurred without significant differences in food intake across groups, suggesting that the enhanced EE was most likely a direct consequence of methionine depletion rather than reduced caloric consumption. Additionally, the increase in lean mass in the CD and HD groups by week 11 suggests a shift toward more metabolically active tissue composition, which may further amplify EE and mitigate fat accumulation. Though methionine‐replete groups displayed similar levels of energy expenditure at all stages regardless of dietary fat composition, prolonged methionine depletion coupled with high‐fat consumption nominally mitigated the effectiveness of methionine restriction. Although not statistically significant, the HD cohort did not demonstrate on average the same robust increase in energy expenditure as the CD group. So while the presentation of heightened energy expenditure resulting from methionine restriction is not novel, these results do offer some early insight into potential metabolic outcomes when juxtaposing methionine depletion in conjunction with long‐term high‐fat intake (Forney, Fang, et al., 2020; Forney, Stone, et al., 2020; Hasek et al., 2010).

Despite clear metabolic improvements, methionine depletion did not confer measurable changes in arterial blood pressure over the 12‐week period. All groups showed gradual increases in blood pressure over time, but no differences were observed between groups based on dietary methionine or fat content. This finding contrasts with the proposed mechanism of maintaining vascular homeostasis within obesogenic conditions via upregulation of the gaseous signaling molecule hydrogen sulfide (H_2_S). Regarding vascular reactivity, preserved PVAT anti‐contractile function across all groups, including those fed a HFD, was unexpected. Previous studies have shown that obesity and HFD feeding impair PVAT‐mediated vasodilation through increased oxidative stress or diminished levels of adiponectin, leptin, and H_2_S (Almabrouk et al., 2014; Xia & Li, 2017). However, in our study, neither HFD nor methionine depletion disrupted PVAT's capacity to suppress norepinephrine (NE)‐induced contractions or to enhance acetylcholine (ACh)‐mediated relaxation. It is possible that the 12‐week duration was insufficient to induce detectable impairments in PVAT function. Further, the relatively younger age or strain of Wistar rats may have contributed to the preserved PVAT responsiveness despite HFD exposure. Alternatively, other mediators responsible for resisting a hypertensive state including adiponectin and leptin may have contributed to the maintained vascular integrity despite an obesogenic state. These adipokines have been shown to activate AMPK, an important modulator between PVAT and the vasculature (Weston et al., 2013).

Furthermore, while methionine depletion had some, albeit random and nominal, effects on the ISR family member ATF4 and its target genes' transcription, these effects were not similarly observed at the protein level. Moreover, CBS and CSE were virtually undetectable in multiple PVAT depots via Western Blot analysis. The only consistent shift in target genes was recorded in the liver, with a HFD maintaining methionine surprisingly resulting in increased CBS transcript and protein levels. As such, it would seem that the failure of dietary modifications to elicit functional differences in PVAT, as noted by unmodified systemic arterial pressure and contractility, is due to the significant lack of H_2_S producing enzymes CBS and CSE within the tissue in male Wistar rats, even when challenged with a HFD. What's more, the failure to note any demonstrable changes in vascular tensions, in addition to our Western Blot results, indicates the limitations of the study in addressing the proposed hypothesis. This is in contrast to the hypertriglyceridemic (HTG) rat, a nonobese rat model of genetic hypertriglyceridemia, which demonstrated higher thoracic PVAT protein expression of the H_2_S producing protein CSE when compared to thoracic PVAT from control Wistar rats (Golas et al., 2022). In another study, where male Wistar rats were made obese by feeding a high‐calorie diet for 3 months, PVAT CSE protein expression was reduced (Bełtowski, 2013).

Limitations of our study include the use of only male rats, which may not account for sex‐specific differences in cardiometabolic physiology. It has been routinely observed in several mammalian models (humans, rodents, etc.) that premenopausal females exhibit a reduced propensity for cardiovascular perturbations such as atherosclerosis relative to males (Patten, 2007). Specifically, in rodents placed on a high‐fat, high‐sugar diet, males demonstrated disruptions in blood pressure and ventricular cardiac output while females remained unaffected (Croft et al., 2024). And so, since our experimental conditions did not manifest cardiovascular dysfunction in male mice, we speculate that females would have similarly maintained arterial pressure and contractile properties, as they are the sex cohort that displays significant innate cardioprotection. Regarding sex‐specific differences in body composition and metabolic effects of a low methionine diet, Forney et al. observed that in C57BL6/J mice, body weight and body composition changes with a low methionine diet were in general similar to those observed in males (Fang et al., 2021). They observed that there were sex differences in young mice on low methionine diets with regards to fat deposition, but in physically mature adult mice, there were comparable decreases in body weight and lean and fat mass in both male and female mice fed a low methionine diet. Additionally, liver transcriptomic changes with the low methionine diet were similar between young and physically mature male and female mice, suggesting that there is little evidence for sex‐specific molecular differences in response to a low methionine diet (Forney, Stone, et al., 2020). While out of the scope of the study described here, future studies must include studying both sexes to properly characterize the cardiovascular implications of a low methionine diet. While a HFD was used with the intent of inducing obesity (as defined by excessive body mass and adiposity), in our male Wistar rat model, a HFD diet did not ultimately result in greater body mass changes compared to age‐matched male Wistar rats fed a control diet. Previous studies have used various perturbations of dietary fat content to induce an obese phenotype in Wistar rats (including but not limited to these refs (Crisostomo et al., 2024; Lasker et al., 2019; Marques et al., 2016)), but there is no standardization regarding diet formulation (i.e., diet vendor, dietary fat content, source of dietary fat, etc.) to consistently induce a reproducible obesity phenotype in various rodent strains. In many of these studies, the “control” diet is a chow pellet that is comprised of unrefined grain mixtures, compared to the purified diet used in the study reported here, which may also account for different rates of body weight gain in rodents as they age (Apolzan & Harris, 2012). Moreover, there is not yet a “clinical” definition for obesity in rodents as there is in humans. In the study presented here, rats fed a HFD consumed a similar number of calories per body mass as the control diet fed group (Figure 2c), suggesting that perhaps the lack of excessive caloric intake in the HFD group may explain the lack of excessive weight gain in these animals. Further studies, either with longer diet administration to generate adiposity/PVAT/vascular changes or the adoption of a diet more in line with a “Western” diet that is high in salt, sucrose, and fat, might better recapitulate the clinical hallmarks of obesity in the male Wistar rat (Corken et al., 2024), but are outside the scope of the study presented here. In addition, we were unable to detect CSE, CBS, or ATF4 proteins in PVAT by Western blot, which limits our ability to correlate changes in transcript levels with protein expression and downstream functional effects. Another limitation is that the tail cuff method, while noninvasive, may lack the sensitivity of telemetry in detecting subtle blood pressure variations (Harrison et al., 2024). Finally, the absence of direct H_2_S quantification in PVAT or systemic circulation prevents definitive conclusions regarding the role of H_2_S bioavailability in mediating vascular outcomes.

Our study has several important implications. First, it reinforces the potent metabolic benefits of methionine restriction, including lean mass preservation and enhanced EE, without requiring caloric restriction. Second, it challenges the assumption that dietary methionine restriction uniformly improves vascular function via enhanced PVAT‐derived H_2_S signaling, at least in early or moderate stages of obesity. These findings highlight a possible dissociation between metabolic improvements and vascular outcomes under methionine‐restricted conditions, warranting further mechanistic exploration. Future studies should explore the time course of methionine restriction on PVAT phenotype and function, including direct assessment of H_2_S levels and oxidative stress markers. Investigating female models and extending dietary interventions to later stages of obesity or comorbid hypertension may also reveal additional insights. Mechanistic studies evaluating CBS and CSE post‐transcriptional regulation and investigating the presence of amino acid response elements (AAREs) on alternative enzymes involved in the metabolism of sulfur‐containing amino acids like cysteine aminotransferase (CAT) and 3‐mercaptopyruvate sulfurtransferase (3‐MST) are likewise warranted.

## 5 CONCLUSION

In conclusion, methionine depletion in male Wistar rats elicited robust metabolic benefits, including reduced fat mass and elevated energy expenditure, but did not alter blood pressure or enhance PVAT‐mediated vascular function. The results of this study provide a necessary first step in investigating previously unexplored avenues to mitigate the vascular dysfunction that accompanies obesogenic diets. We provide insight into the expression of the ISR member ATF4, as well as its target genes within multiple PVAT depots that will inform future pursuits to tackle the issue of PVAT functional disruption in the context of obesity. These results highlight the complexity of dietary interventions in cardiovascular health and emphasize the need for further investigation into the temporal and mechanistic interplay between metabolic and vascular responses to methionine restriction.

## FUNDING INFORMATION

This research was supported by funding from USDA ARS 6026‐10700‐001‐000D and a University of Arkansas for Medical Sciences Sturgis Foundation Grant Award.

## CONFLICT OF INTEREST STATEMENT

The authors declare that they have no known competing financial interests or personal relationships that could have appeared to influence the work reported in this paper.

## ETHICS STATEMENT

The animal study protocol was approved by the University of Arkansas for Medical Sciences Institutional Animal Care and Use Committee (IPROTO202400000124, approved 10/31/2024).

## Supporting information


Appendix S1.

## Data Availability

The dataset outlined in this manuscript is available from the corresponding author upon request.
