# Supplementary material for: Dietary methionine depletion and hydrogen sulfide‐producing genes in perivascular adipose tissue of male Wistar rats
Source: Physiol Rep. 2025 Dec 19;13(24):e70702. doi: 10.14814/phy2.70702 (PMC12717448; doi:10.14814/phy2.70702)
Supplement: Supplementary file 1 — Appendix S1. [file PHY2-13-e70702-s001.docx]

Supplementary Material

| Real-time qPCR primer | Sequence (5’-3’) |
| --- | --- |
| ATF4 (Forward) | TCAGAATGGCTGGCTATGGAT |
| ATF4 (Reverse) | TTCAGGTCCATTTTCTCCAACA |
| CBS (Forward) | ACCACACAGTGCCGACAAAAT |
| CBS (Reverse) | TGCATTCTTGGAGATCCTGTTG |
| CSE (Forward) | ATCCGGATGGAGAAACACTTCA |
| CSE (Reverse) | TGAGACGGTAGCCCAGGATAA |
| UCP1 (Forward) | AAGGCCAGGCTTCCAGTACTATT |
| UCP1 (Reverse | GGCAGACCGCTGTACAGTTTC |
| Leptin (Forward) | TCACCGGTTTGGACTTCATTC |
| Leptin (Reverse) | GGCAAGCTGGTGAGGATCTG |
| β-actin (Forward) | AAGGCCAACCGTGAAAAGATG |
| β-actin (Reverse) | CCAGAGGCATACAGGGACAAC |

**Supplementary Table 1.** Real-time quantitative PCR primer sequences
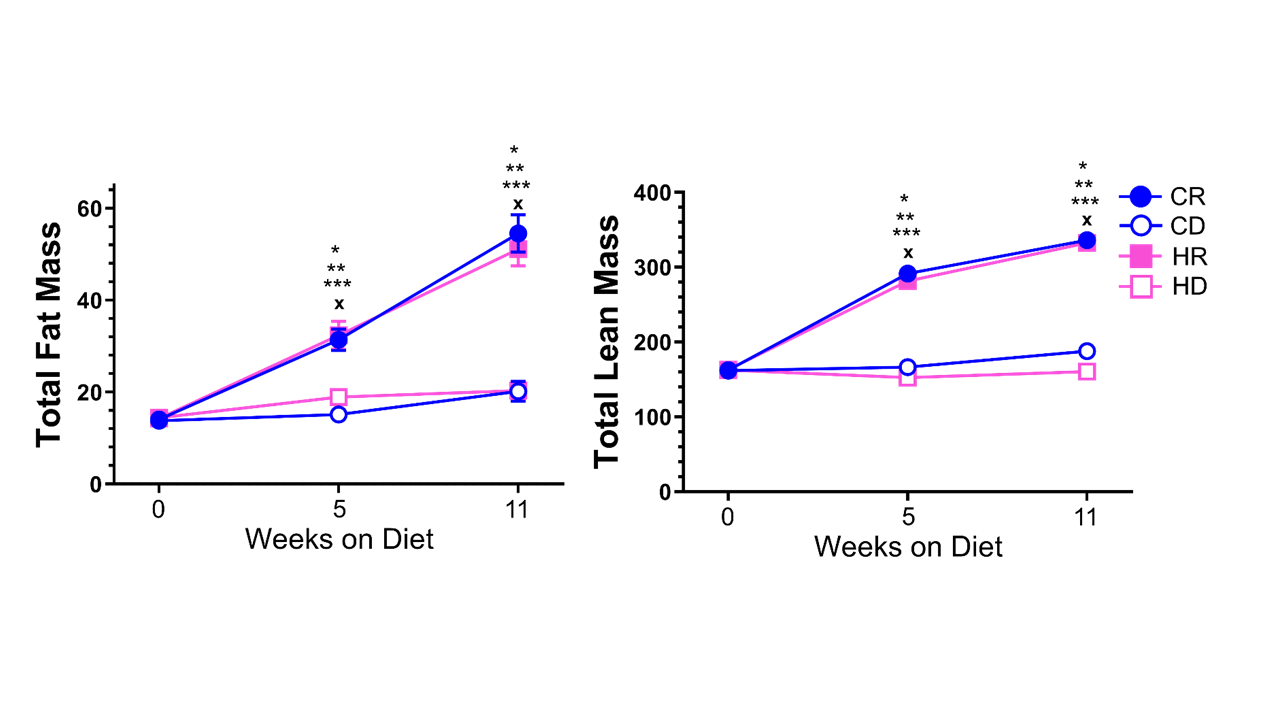
**Supplementary Figure 1.** Total fat and lean mass quantification for all groups across multiple time points. Total fat mass **A**) was assessed at baseline and at 5 and 11 weeks of diet administration [5 weeks - *P*(value) < 0.001 - *CR v CD; *P*(value) = 0.0010 - **CR v HR; *P*(value) < 0.0001 - ***CD v HR; *P*(value) = 0.0003 – ^X^HR v HD. 11 Weeks – *P*(value) < 0.0001 *CR v CD; *P*(value) < 0.0001 **CR v HD; *P*(value) < 0.0001 ***CD v HR; *P*(value) < 0.0001 ^X^HR v HD] in addition to total lean mass **B**) [5 weeks - *P*(value) < 0.0001 - *CR v CD; *P*(value) < 0.0001 - **CR v HD; *P*(value) < 0.0001 - ***CD v HR; *P*(value) < 0.0001 – ^X^HR v HD. 11 Weeks – *P*(value) < 0.0001 - *CR v CD; *P*(value) < 0.0001 - **CR v HD; *P*(value) < 0.0001 - ***CD v HR; *P*(value) < 0.0001 – ^X^HR v HD]. N = 10 per group.

**A)**

**B)**


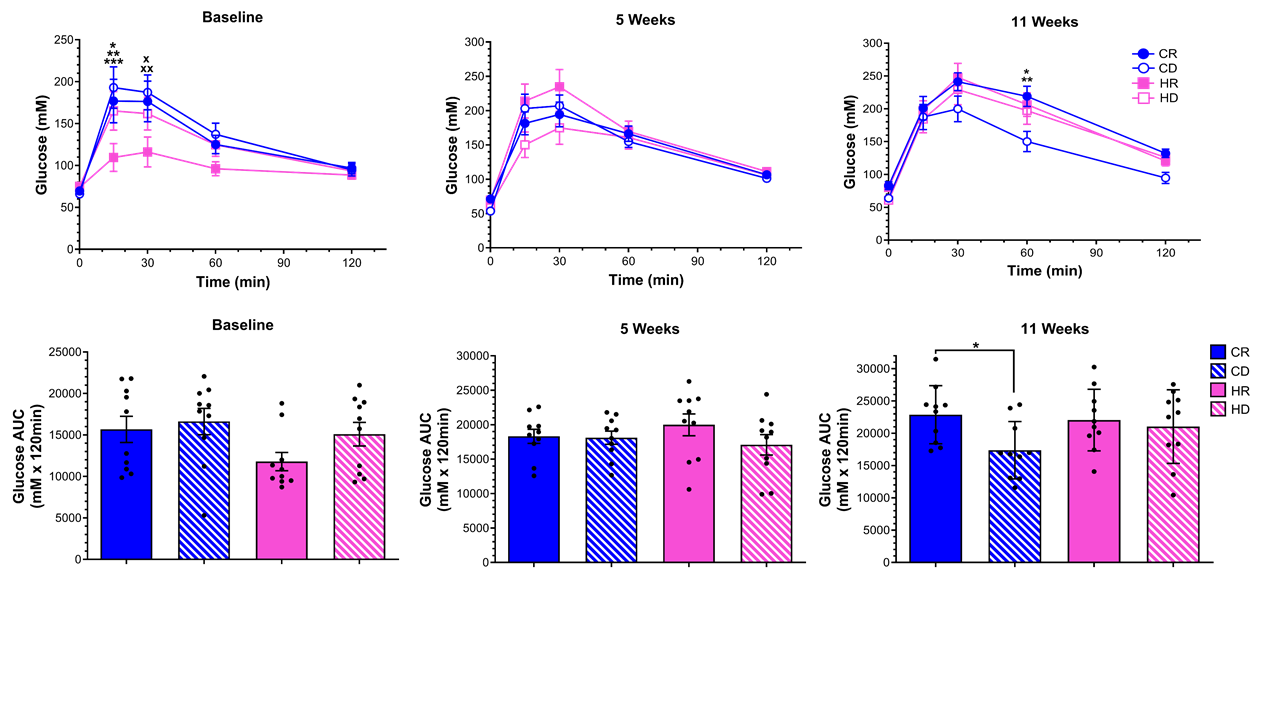


**A)**

**B)**

**C)**

**D)**

**E)**

**F)**

**Supplementary Figure 2.** Glucose tolerance assessment. Average blood glucose levels were obtained for all intervention [Baseline - *P*(value) = 0.0101 - *CR v HR; *P*(value) = 0.0008 - **CD v HR; *P*(value) = 0.0478 - ***HR v HD; *P*(value) = 0.0267 – ^X^CR v HR; *P*(value) = 0.0058 – ^XX^CD v HR. 11 Weeks – *P*(value) = 0.0071 *CR v CD; *P*(value) = 0.0408 **CD v HR]. Corresponding area under the curve (AUC) average values of blood glucose concentrations for samples obtained **D)** at baseline, **E)** 5 weeks and **F)** 11 weeks [**P*(value = 0.0477] after diet administration N = 10 per group.


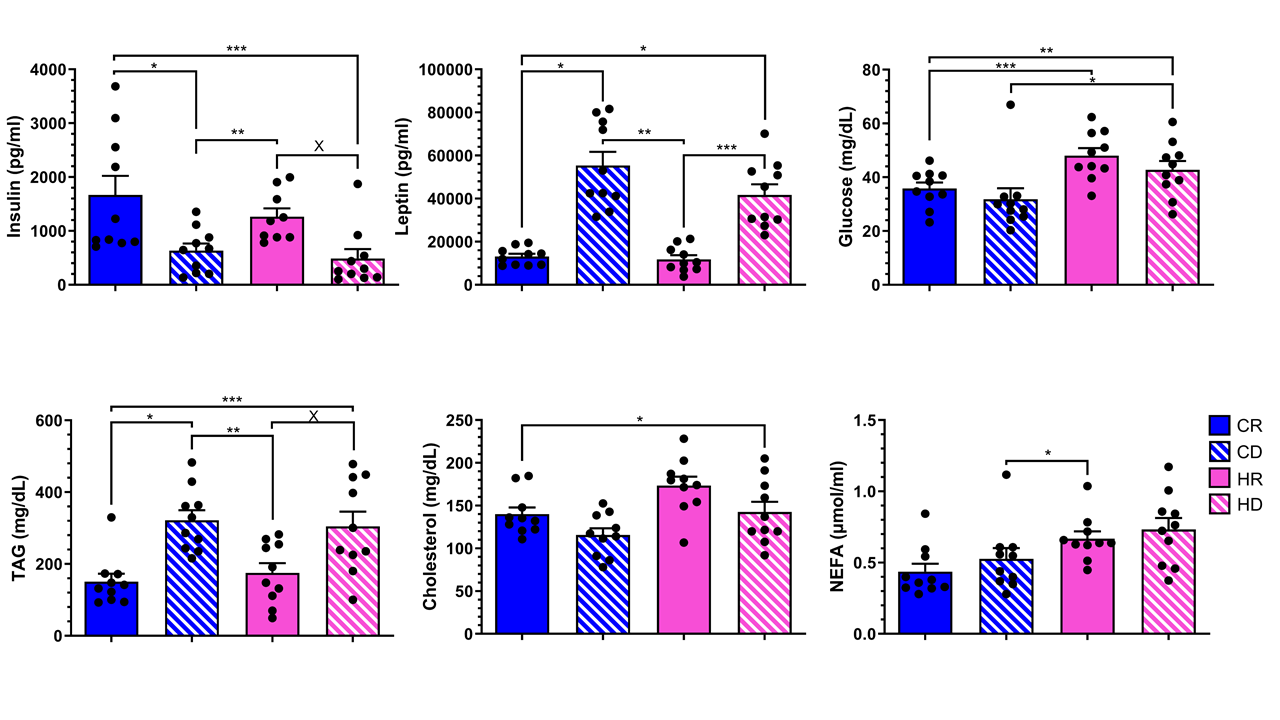


**A)**

**B)**

**C)**

**D)**

**E)**

**F)**

**Supplementary Figure 3.** Serum analysis of metabolic analytes following 11 weeks of diet administration. The average serum levels of insulin **A)** [**P*(value) = 0.0013; ***P*(value) = 0.0266; ****P*(value) = 0.0003; ^X^*P*(value) = 0.0067], leptin **B)** [**P*(value) < 0.0001; ***P*(value) = 0.0005; ****P*(value) = 0.0003], glucose **C)** [**P*(value) = 0.0089; ***P*(value) = 0.0005; ****P*(value) = 0.0204], TAG **D)** [**P*(value) = 0.0020; ***P*(value) = 0.0055; ****P*(value) = 0.0087; ^X^*P*(value) = 0.0229], cholesterol **E)** [**P*(value) = 0.0030] and NEFA **F)** [**P*(value) = 0.0250] are represented above for all groups. N = 10 per group.

**
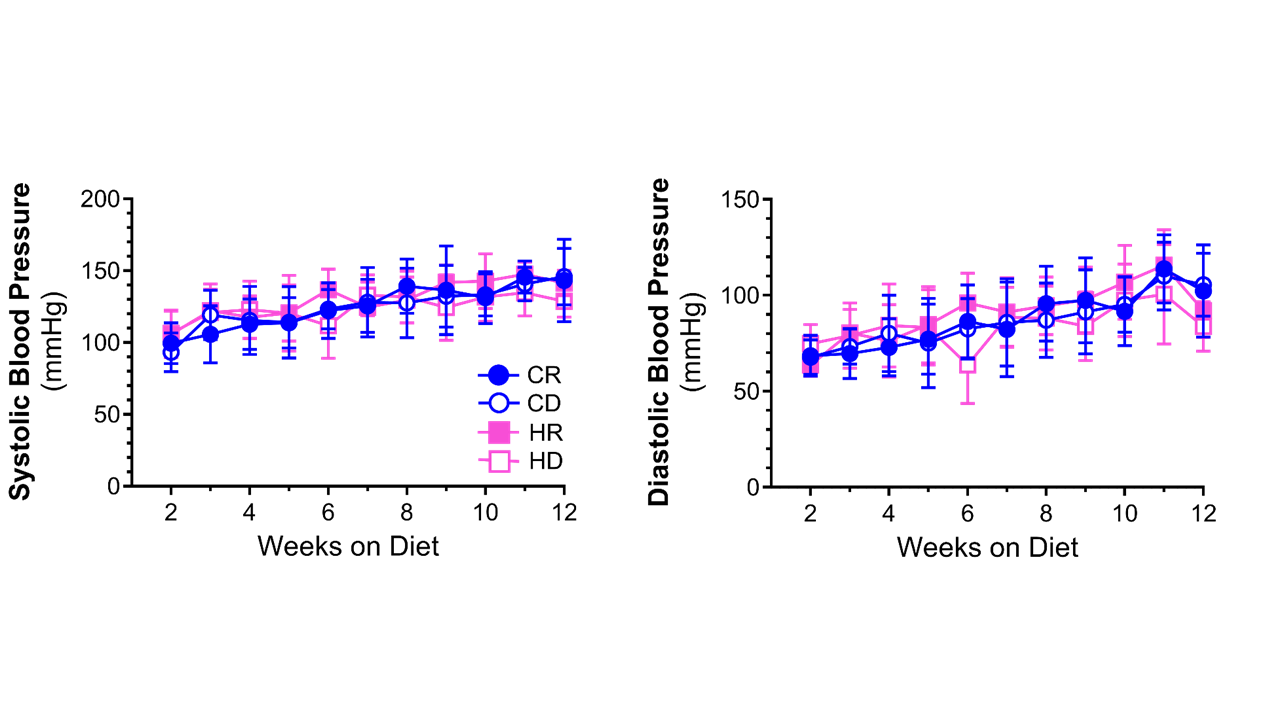
Supplementary Figure 4.** Systolic **A**) and diastolic **B**) blood pressure measurements were conducted beginning at two weeks of dietary administration. Assessment was continued weekly until the study’s conclusion at 12 weeks of diet administration. No statistical differences were observed between groups throughout the duration of the study. N = 10 per group.

**A)**

**B)**


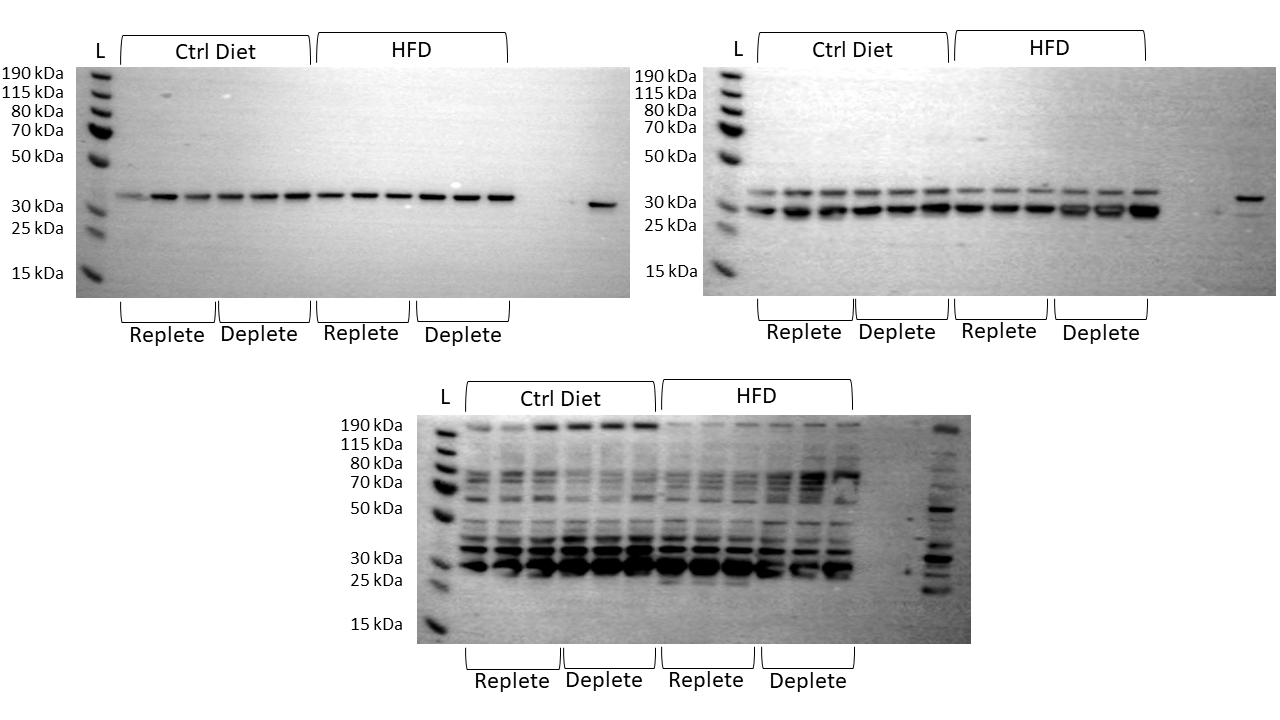
**Supplementary Figure 5.** Western blotting of aPVAT lysates. The blot was first stained for GAPDH which is visible for all samples **A**). The blot was reused to stain for ATF4, CBS and CSE which were not observed. Next the same blot was stained for UCP1 **B**) which was visible for all samples. Finally the blot was stained for Leptin **C**) which was not present for aPVAT samples but detected in the WAT control lane (far right lane).

**A)**

**B)**

**C)**


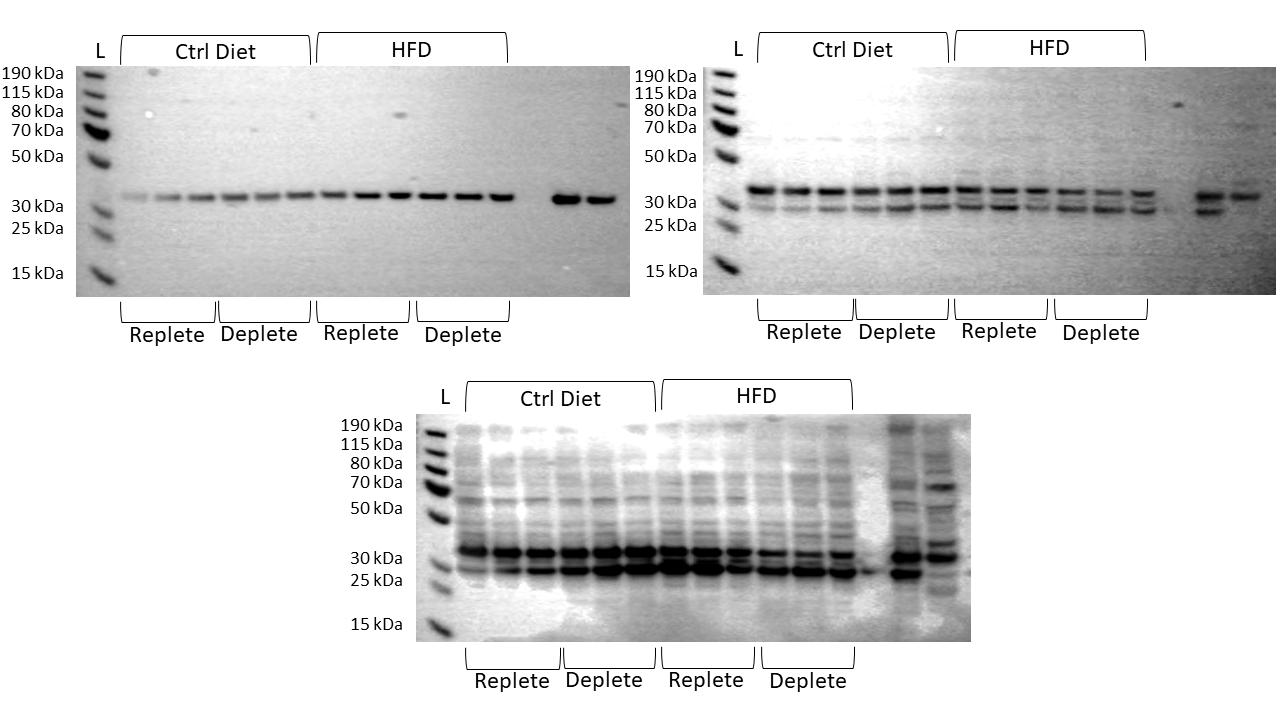


**A)**

**B)**

**C)**

**Supplementary Figure 6.** Western blotting of tPVAT lysates. The blot was first stained for GAPDH which is visible for all samples **A**). The blot was reused to stain for ATF4, CBS and CSE which were not observed. Next the same blot was stained for UCP1 **B**) which was visible for all samples. Finally the blot was stained for Leptin **C**) which was not present for aPVAT samples but faintly detected in the WAT control lane (far right lane).


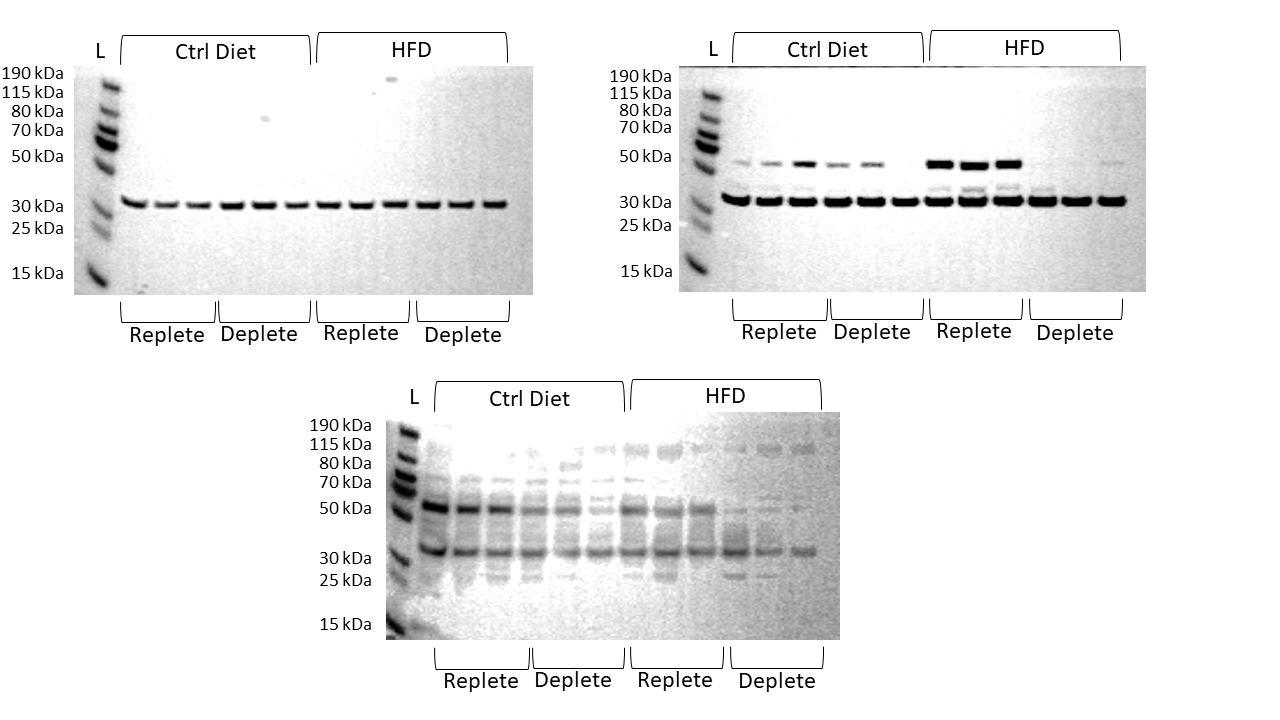


**A)**

**B)**

**C)**

**Supplementary Figure 7.** Western blotting of liver lysates. The blot was first stained for GAPDH which is visible for all samples **A**). The blot was reused to stain for CBS **B**) which was visible in the majority of all groups with the exception of HFD deplete. Next the same blot was stained for ATF4 **C**) which was fainly detected.

**Supplementary Figure 8.** Western blotting of liver lysates. Using a new blot, GAPDH was stained for and detected in samples **A**). The blot was reused to stain for CSE **B**) which was visible in all groups
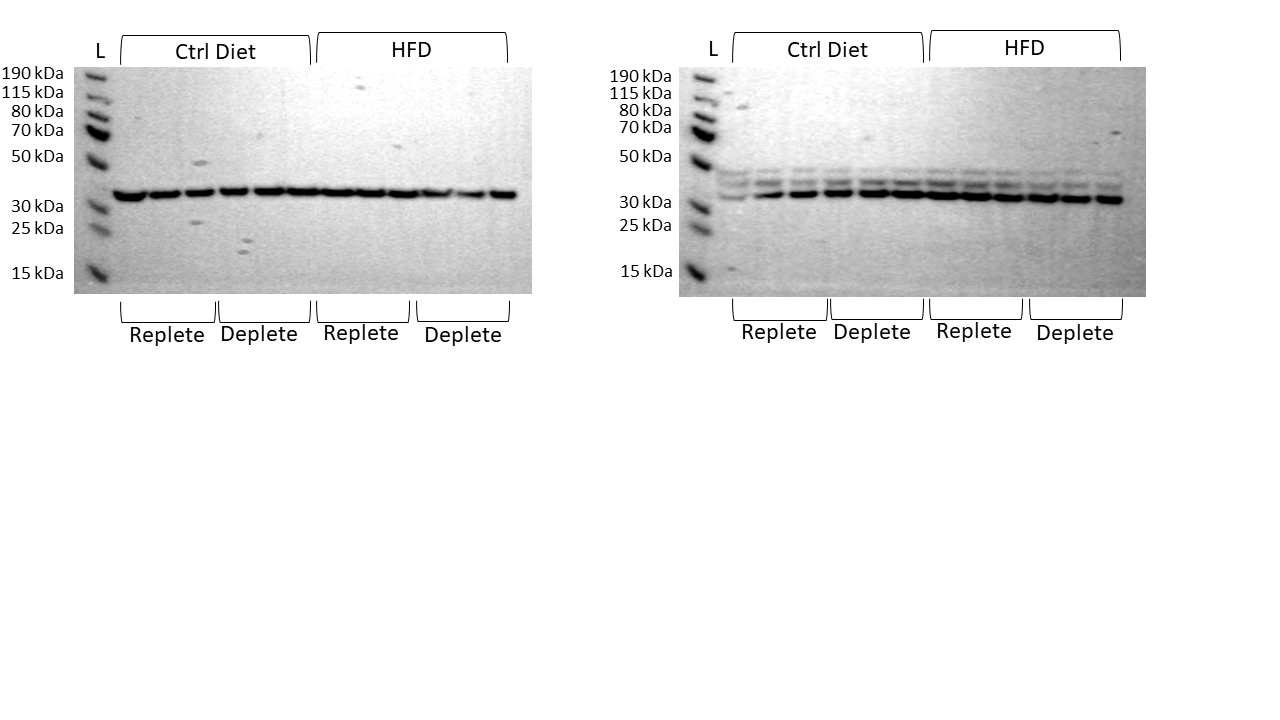
.

**A)**

**B)**
